# Gesture Imitation Performance and Visual Exploration in Young Children with Autism Spectrum Disorder

**DOI:** 10.1007/s10803-024-06595-w

**Published:** 2024-10-15

**Authors:** Kenza Latrèche, Nada Kojovic, Irène Pittet, Shreyasvi Natraj, Martina Franchini, Isabel M. Smith, Marie Schaer

**Affiliations:** 1 Department of Psychiatry, Faculty of Medicine, University of Geneva, 1206 Geneva, Switzerland; 2 Fondation Pôle Autisme, 1204 Geneva, Switzerland; 3 Department of Pediatrics, Dalhousie University, IWK Health Centre, 5850 University Avenue, P. O. Box 9700, Halifax, NS B3K 6R8 Canada; 4 Autism Research Centre, IWK Health Centre, 4th Floor Link Building, 5850/5980 University Avenue, P.O. Box 9700, Halifax, NS B3K 6R8 Canada

**Keywords:** Autism spectrum disorders, Imitative behavior, Eye-tracking, Visual attention, Motor execution

## Abstract

Most studies in children with autism spectrum disorder (ASD) support a decrement in imitation performance. Factors related to visual attention and motor execution have been proposed to explain this phenomenon in ASD. However, studies investigated imitation with various methods, leading to inconsistent findings. Here, we examine imitation performance related to visual attention and motor execution. We focused on the imitation of meaningful and meaningless gestures, consistently reported as more affected than imitation of actions with objects in ASD. The imitation eye-tracking task consisted of a video of an actor demonstrating gestures and prompting children to imitate them. The demonstrations comprised meaningful and meaningless hand gestures, and meaningless facial gestures. We measured the fixation duration to the actor’ face during child-directed speech and gesture demonstrations. We video-recorded children to assess their performance. Our sample comprised 100 participants (3.55 ± 1.11 years old), including 84 children with ASD. The ASD and typically developing groups displayed the same visual attention toward gesture demonstrations, although children with ASD spent less time looking at the face during facial stimuli. Visual exploration of actors’ gestures did not influence imitation performance. Imitation of meaningful gestures was associated with less severe autistic symptoms, whereas imitation of meaningless gestures was correlated with higher non-verbal cognitive and fine-motor skills. These findings contribute to a better understanding of the complexity of imitation. We delineated the distinct nature of imitation of meaningful and meaningless gestures in children with ASD. We discuss clinical implications in relation to assessment and intervention programs.

**Supplementary Information:**

The online version contains supplementary material available at 10.1007/s10803-024-06595-w.

## Introduction

The ability to imitate develops from a very early age (Jones & Herbert, 2009; Vivanti & Hamilton, 2014). Studies have reported that neonatal infants can imitate facial, oral, and hand gestures (Anisfeld, 1996; Meltzoff & Moore, 1989; Nagy et al., 2020). In subsequent development, imitation consolidates during unstructured dyadic interactions (Nielsen et al., 2006; Rogers et al., 2003; Vivanti & Hamilton, 2014). Over the first two years of life imitation behaviors become progressively more complex, starting from vocalizations and simple actions directed on objects (e.g., banging a toy) and extending to meaningful and meaningless manual and facial gestures (Young et al., 2011). The meaning and familiarity of gestures are parameters that influence the propensity and ability to imitate. Indeed, imitation of familiar, meaningful hand and facial gestures (e.g., applause, smiling) is mastered earlier than the imitation of meaningless gestures (Vivanti & Hamilton, 2014; Young et al., 2011).

The important role of imitation in development has been consistently recognized. Imitation appears as an essential tool for social learning and acquiring new skills during early childhood (Rogers & Pennington, 1991; Vivanti & Hamilton, 2014). Imitation promotes crucial developmental processes, including language (Charman et al., 2003; Nielsen et al., 2006) and social skills (Vivanti & Hamilton, 2014; Young et al., 2011), as well as theory of mind (Rogers & Pennington, 1991) and joint attention (Ezell et al., 2012). Such early language and social abilities are core areas of differences associated with autism spectrum disorder (ASD) (Baron-Cohen, 2000; Boucher, 2012; Dawson et al., 2002), which is defined by difficulties in social communication and interaction and the presence of repetitive and restricted behaviors (American Psychiatric Association, 2013). Given its implication in early social development, imitation has generated much interest in autism research (Rogers & Pennington, 1991; Smith & Bryson, 1994; Vivanti & Hamilton, 2014; Young et al., 2011).

Imitation research in ASD has mainly focused on two types of imitation, namely spontaneous imitation and elicited imitation performance (Vivanti & Hamilton, 2014). Spontaneous imitation is assessed with naturalistic observations or parental reports. Most studies have reported lower frequency of spontaneous imitation in children with ASD (Colombi et al., 2009; Ingersoll, 2008), whereas others have not (Charman & Baron-Cohen, 1994).The present paper focuses on elicited imitation, a paradigm in which individuals are explicitly instructed to imitate an action (Vivanti & Hamilton, 2014). Previous studies have revealed disruptions in imitation performance in siblings at an increased likelihood for autism and in autistic children. A meta-analysis (Edwards, 2014) further revealed that imitation deficits in autistic individuals remained significant regardless of the format of the imitation tasks (i.e., live or screen-based). Focusing on the Autism Observation Scale for Infants (AOSI; Bryson et al., 2008), which includes an elicited imitation task, Zwaigenbaum et al. (2005) highlighted that by 12 months of age, siblings of children with ASD who were later diagnosed with ASD showed lower imitation performance than those who were not diagnosed. Young et al. (2011) used a 10-item imitation battery comprising manual and facial gestures and actions on objects in a longitudinal sample of siblings at an increased likelihood for ASD and TD infants aged 12 to 24 months. The authors showed that infants who later developed ASD exhibited delayed imitation development. We also recently showed that toddlers with ASD had difficulties in imitating both actions on objects and gestures (Pittet et al., 2022).

Several explanations have been proposed to explain imitation difficulties in ASD. However, the mechanisms remain unclear. Vivanti and Hamilton (2014) proposed that attentional, social, and executive factors may all play a key role in imitation in ASD. Attentional difficulties correspond to a lack of attention to the demonstrated actions, whereas social difficulties are involved in the processing and understanding of social information, and executive factors relate to difficulties in motor execution and performance of actions (Vivanti et al., 2014). To examine these factors, Vivanti and collaborators developed innovative imitation tasks combining eye-tracking technology and video recordings of children’s imitative behavior of meaningless gestures, and meaningful actions with or without objects (Vivanti et al., 2008, 2011, 2014). In addition, Akin-Bulbul and Ozdemir (2022) recently developed an eye-tracking paradigm combined with video recordings, and studied six types of imitation (meaningful and meaningless gestures, vocalizations, and actions on objects) in toddlers with ASD, developmental delay (DD), and TD matched on cognitive level. These studies (Akin-Bulbul & Ozdemir, 2022; Vivanti et al., 2008, 2011, 2014) found contrasting results. Although the number of fixations to the demonstrator’s face did not differ between ASD and TD groups, participants with ASD showed poorer imitation skills. Vivanti et al. (2008) studied children with ASD and their TD peers who were matched for chronological age and cognitive level. Both groups attended similarly to the actions and to face regions during meaningless gestures, but the ASD group imitated less accurately. Similarly, Vivanti et al. (2011) showed that the number of fixations to the demonstrator’s face did not differ between ASD and TD groups matched for chronological age and cognitive level. However, participants with ASD showed poorer imitation skills. Additionally, no significant correlation between imitation performance and visual exploration was found in either group. In a subsequent study using a different task, the authors (Vivanti et al., 2014) studied lower functioning preschoolers with ASD, which revealed that they spent less time looking at the demonstrator’s face and also showed poorer imitation skills than children with TD and developmental delay (DD). Akin-Bulbul and Ozdemir (Akin-Bulbul & Ozdemir, 2022) showed that toddlers with ASD displayed diminished attention to the face and action regions. Moreover, children with ASD and DD showed lower performance than TD children for all types of imitation, suggesting that no imitation type was preserved (Akin-Bulbul & Ozdemir, 2022). In addition, participants with DD showed better imitation of meaningful gestures than participants with ASD.

Several elements may be involved in these conflicting findings (Akin-Bulbul & Ozdemir, 2022; Vivanti et al., 2008, 2011, 2014). Firstly, these inconsistencies may result from heterogeneity in age and intellectual abilities across participants with ASD (i.e., from toddlers to adolescents with no intellectual disability). Secondly, the inclusion of different types of imitation (namely meaningful and meaningless actions on objects, manual and facial gestures, and vocalizations) may contribute to contrasting findings given that imitation with objects appears earlier and is easier for children with ASD compared to the imitation of gestures, which is more complex and may depend on familiarity (Ingersoll & Meyer, 2011; Vivanti & Hamilton, 2014). Indeed, research on imitation performance with older children, adolescents and adults with ASD tends to report differences that specifically affect meaningless gestures (Carmo et al., 2013; Vivanti & Hamilton, 2014). Additionally, Stone and colleagues (Stone et al., 1997) showed that while the imitation of body and facial gestures predicted speech development, imitation of actions with objects was associated with play, suggesting that imitation is not a unitary skill (Rogers et al., 2003; Stone et al., 1997). It is established that imitation of gestures is more difficult than imitation of actions with objects in ASD (Ingersoll & Meyer, 2011; Smith & Bryson, 1994, 2007; Zachor et al., 2010). Nevertheless, to our knowledge, no study has yet examined meaningful and meaningless gesture imitation in a younger sample of children with ASD.

Thus, the present study aims to investigate the imitation of hand and facial gestures jointly with visual attention processes. Using an eye-tracking paradigm combined with video recording, we compared three types of gestures, namely meaningful hand gestures, meaningless hand gestures, and meaningless facial gestures. To understand the processes behind imitation behavior better, we adopted an innovative approach and concentrated on factors relating to visual attention and imitation performance in a sample of 84 children with ASD (aged 3.55 ± 1.11 years) and 16 TD children (aged 3.31 ± 1.17 years). We defined the visual attention factor as visual exploration toward the demonstration of gestures and to the actors’ social cues (i.e., when they asked the child to look at them and to imitate). Additionally, we explored the association between the children’s visual exploration and their imitative behavior. As to the imitation performance factor, we investigated whether individual characteristics of children (e.g., age, developmental skills, autistic symptoms) were related to imitation performance. We hypothesized that young children with ASD exhibiting more attention towards the gesture demonstrations and the actors’ faces during social cues would display more accurate imitation. Furthermore, in line with previous literature (Akin-Bulbul & Ozdemir, 2022; Pittet et al., 2022; Vivanti & Hamilton, 2014; Williams et al., 2004; Zwaigenbaum et al., 2005), we hypothesized that children with ASD would show diminished attention to the demonstrators’ face and perform more poorly than their TD peers and that imitation performance would be positively associated with age and developmental skills. Such results would help to unravel the roles of visual attention and imitation performance and their contributions to imitation behavior. Given the importance of imitation in early development, these results would further contribute to targeting and adapting existing interventions for ASD.

## Methods

### Participants

Children participating in this study were enrolled in the Geneva Autism Cohort, an ongoing longitudinal study that started in 2012 (for a description, see Franchini et al., 2016; Robain et al., 2020). In the present study, we administered our eye-tracking imitation task to 100 participants younger than 6 years, among which were 84 children with ASD (14 females, aged 3.55 ± 1.11 years) and 16 TD children (7 females, aged 3.31 ± 1.17 years) (see Additional file 1). The two groups did not significantly differ by age (p = 0.433). Children with ASD were recruited through local clinics specializing in child psychiatry and from family associations. Diagnoses of ASD were confirmed using the Autism Diagnostic Observation Schedule, second edition (ADOS-2) (Lord et al., 2012). The TD children were recruited through word of mouth and were also assessed with the ADOS-2 (Lord et al., 2012) to confirm the absence of autistic symptoms. For the TD group, inclusion in the present study also required no developmental concerns about the child and no first-degree relative with ASD. The experimental protocol was approved by the Ethics Committee of the Faculty of Medicine of the University of Geneva, and we obtained informed consent from the parents of all participants.

To explore both visual attention and imitation performance during our imitation task, we collected eye-tracking and behavioral data. Among the 100 children who were administered the eye-tracking task, inclusion of participants was done separately for eye gaze and behavior data. Regarding eye-tracking, only children who attended to the screen at least 50% of each of the three sections of the task were included in the analyses, which led to different samples for each of the three conditions (Table 1). Of the 100 children, we excluded 7 children with ASD (5 males and 2 females) who did not meet the 50% criterion, for any of the three conditions. As for behavioral data, we acquired video recordings of the children during the task using The Observer XT™ 14.2 software for later behavioral annotation. Due to technical reasons related to video recording, we were able to include 69 children with ASD and 13 with TD with complete video recording of the tasks (Table 1). In total, we obtained three samples relating to the visual attention factor (i.e., eye-tracking data) and one sample for the imitation performance factor (i.e., video recording data). In these four samples, children with ASD and their TD peers did not significantly differ from the initial sample of 100 children in terms of age, developmental skills (i.e., verbal and non-verbal skills), and autistic symptoms (p > 0.05). Given the small TD sample, it only served for initial between-group comparisons.

**Table 1:** Cross-sectional sample demographics

	Visual attention factor									Imitation performance		
	Meaningful hand gesture (*N* = 83)			Meaningless hand gesture (*N* = 70)			Meaningless facial gesture (*N* = 72)			Video recording (*N* = 82)		
	ASD *n* = 68 (11F)	TD *n* = 15 (7F)	p	ASD *n* = 55 (6F)	TD *n* = 15 (7F)	p	ASD *n* = 57 (10F)	TD *n* = 15 (7F)	p	ASD *n* = 69 (10F)	TD *n* = 13 (6F)	p
Age *M (*± *SD)*	3.47 (± 1.13)	3.33 (± 1.21)	0.653	3.62 (± 1.15)	3.30 (± 1.21)	0.346	3.58 (± 1.10)	3.33 (± 1.21)	0.448	3.50 (± 1.13)	3.11 (± 1.17)	0.307
MSEL total	80.89 (± 20.25)	114.5 (± 14.55)	**< 0.001**	82.01 (± 19.73)	113.7 (± 13.7)	**< 0.001**	82.74 (± 20.28)	114.5 (± 14.55)	**< 0.001**	78.76 (± 22.38)	115.6 (± 15.20)	**< 0.001**
MSEL verbal	73.01 (± 24.60)	107.90 (± 13.37)	** < 0.001**	74.22 (± 23.30)	107.10 (± 11.82)	** < 0.001**	74.57 (± 24.80)	107.90 (± 13.37)	** < 0.001**	69.97 (± 26.97)	109.30 (± 15.10)	** < 0.001**
MSEL non-verbal	88.76 (± 18.31)	122.00 (± 19.88)	** < 0.001**	89.80 (± 18.76)	120.80 (± 19.90)	** < 0.001**	90.91 (± 18.33)	122.00 (± 19.88)	** < 0.001**	87.55 (± 19.93)	123.30 (± 21.05)	** < 0.001**
ADOS total	6.83 (± 1.67)	1.20 (± 0.68)	**< 0.001**	6.57 (± 1.67)	1.29 (± 0.61)	** < 0.001**	6.75 (± 1.71)	1.20 (± 0.68)	** < 0.001**	6.85 (± 1.83)	1.083 (± 0.83)	** < 0.001**

MSEL Total, Mullen Scales of Early Learning, Total Developmental Quotient. MSEL Verbal, Mullen Scales of Early Learning, Verbal Developmental Quotient, MSEL Non-verbal, Mullen Scales of Early Learning, Non-verbal Developmental Quotient. ADOS-2 Total, Autism Diagnostic Observation Schedule, 2nd edition, Total Severity Score

Bold Values indicate statistical significance (*p* > 0.05)

For all participants, we obtained measures of autistic symptoms, and developmental and adaptive skills. The ADOS-2 (Lord et al., 2012) is the gold-standard evaluation that quantifies autistic symptoms in social communication, social interaction, play, and repetitive behaviors. To compare the scores across the different modules of the ADOS-2, we used the ADOS-2 Calibrated Severity Score (Esler et al., 2015; Gotham et al., 2009) for the Social Affect (SA Severity Score) and Restricted and Repetitive Behaviors (RRB Severity Score) domains and for Total Severity Score (Esler et al., 2015; Hus et al., 2014). The ASD group’s severity scores corresponded to a moderate level of symptoms (ranging from 6.57 to 6.85 across the four eye-tracking and behavior samples, Table 1).

We administered the Mullen Scales of Early Learning (MSEL) (Mullen, 1995), which measures development in two non-verbal and two verbal domains, namely the subscales Fine Motor (FM), Visual Reception (VR), Receptive Language (RL) and Expressive Language (EL). To compute developmental quotient (DQ) scores for each scale, we divided the developmental age by the chronological age and multiplied by 100 (Lord et al., 2006). The MSEL Total DQ, comprising all domains, was significantly lower in the ASD group than in the TD group (Table 1).

Finally, we included a measure of adaptive behavior with the Vineland Adaptive Behavior Scales, Second edition (VABS-II) (Sparrow et al., 2005). The VABS-II consists of an interview with the child’s parent and explores adaptive functioning across four domains: Communication (Com), Daily Living Skills (Dai), Socialization (Soc), and Motor Skills (Mot). We used the standard scores for each of the four domains.

### Stimuli and Apparatus

**Fig. 1:**
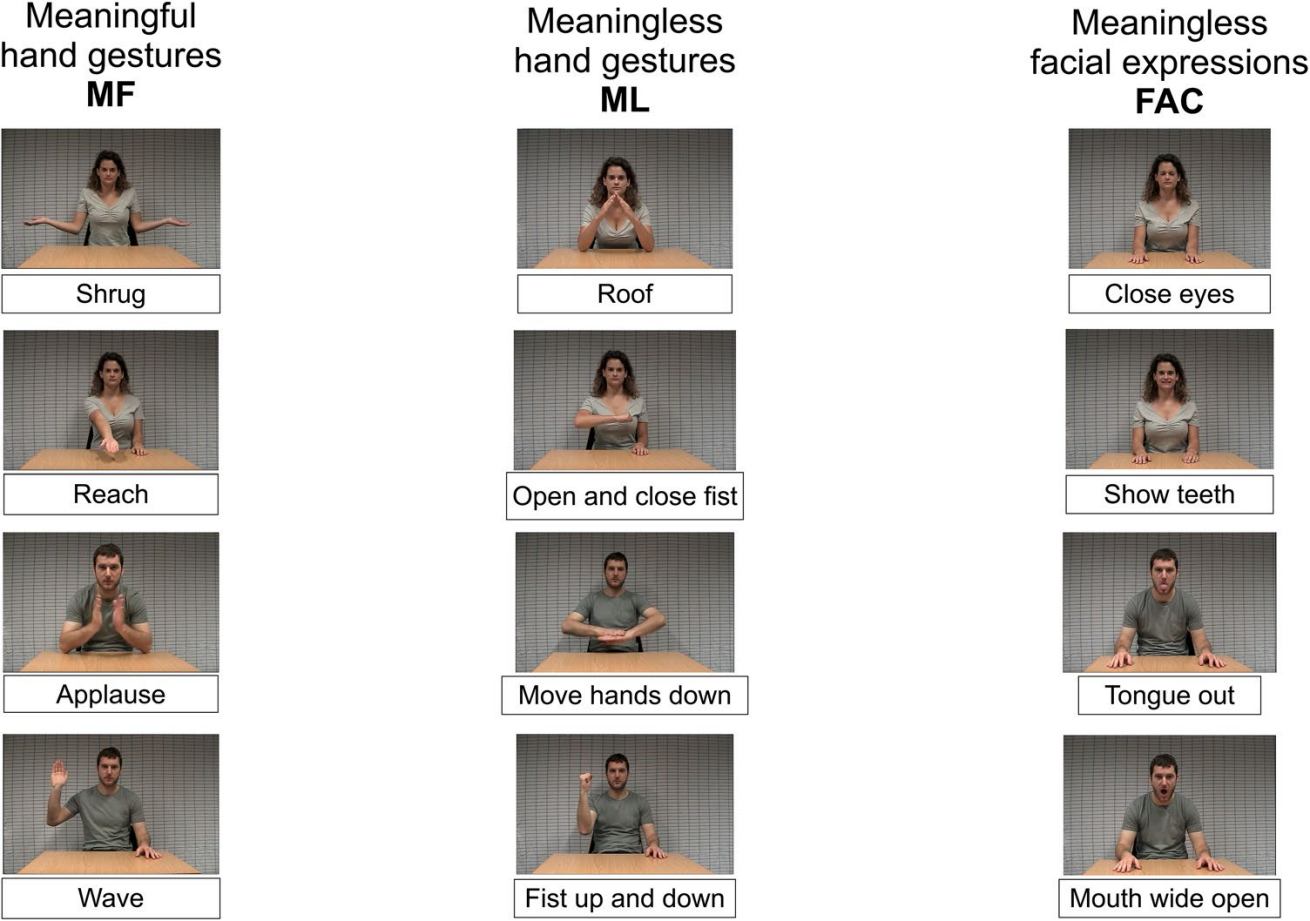
Stimuli of the eye-tracking imitation task. The 12 stimuli of the task comprised four meaningful hand gestures (MF), four meaningless hand gestures (ML), and 4 meaningless facial gestures (FAC). Each participant watched a total of three gestures, one of each type

For the eye-tracking imitation task, each participant was presented with three videos, one meaningful (MF) hand gesture, one meaningless (ML) hand gesture, and lastly one meaningless facial (FAC) gesture. Each of the three videos is randomly chosen among 4 possible videos in the given condition (see Fig. 1). A total of 12 videos displaying one actor or one actress was used for his task. In each of the 12 videos, one female or male actor demonstrated one gesture while sitting at a table, with no stimuli other than a gray rectangular grid in the background (see Fig. 1). The 12 gestures were of three types: four meaningful (MF) hand gestures, four meaningless (ML) hand gestures, and four meaningless facial (FAC) gestures, as shown in Fig. 1. The male and female actors demonstrated six gestures each. The 12 clips were randomized, yielding 64 possible conditions.

**Fig. 2:**
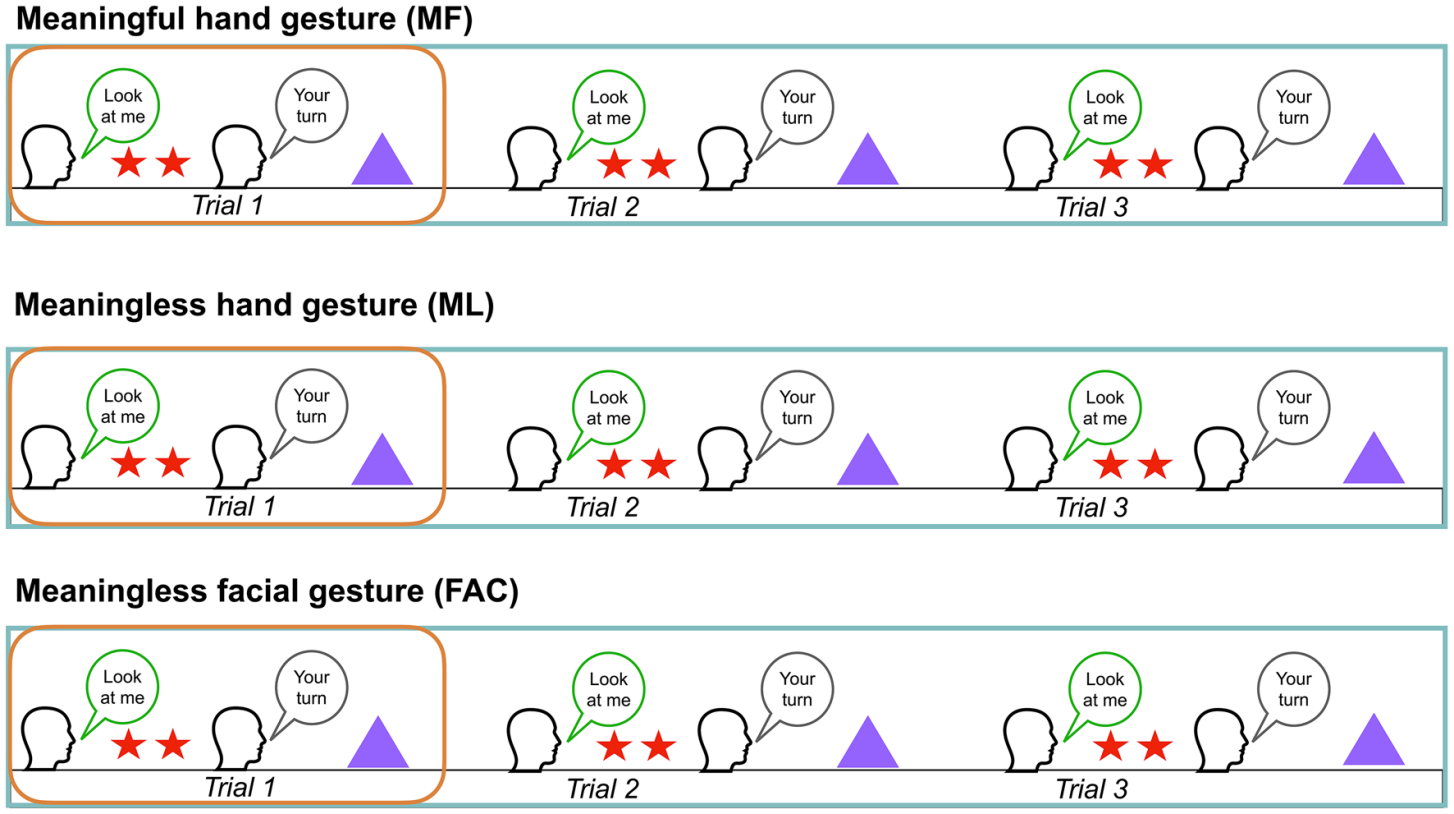
Structure of the eye-tracking imitation task. The task is divided into three parts following the same structure: one meaningful hand gesture (MF), one meaningless hand gesture (ML), and one meaningless facial gesture (FAC). Each part (represented by the blue rectangle) is divided into three trials (represented by the orange section) and begins with an actor asking the child to attend (e.g., “Look at me”) before performing two demonstrations of a gesture (represented as red stars in the figure). After the demonstrations, the actor invites the child to imitate (e.g., “It is your turn”). Then the child is given an opportunity to imitate (as represented by the purple triangle) while the actor remains still. The orange section is repeated three times in total

All gestures were filmed following the same structure (see Fig. 2). At the beginning, the actor attracts the child’s attention by saying (in French) “Look at me”, “Look at what I am doing”, or “Look carefully” with a neutral tone of voice. The actor then silently demonstrates the gesture twice. The demonstrations end with the actor saying, “Now it is your turn” or “You do it” and pausing to give the child the first opportunity to imitate the gesture. During this 5-s pause, the actor looks at the camera with a neutral facial expression and does not talk or move. Since our stimuli were pre-recorded, all participants watched the same video-clips whether they imitated or not. After the 5-s pause, the actor engages twice more in the same procedure: attracting the child’s attention, demonstrating the gesture twice, and pausing to give the child an opportunity to imitate. For each of the three gesture types, the child saw six demonstrations and had three opportunities to imitate. Thus, they were expected to imitate nine times in total.

When designing the eye-tracking task, we purposely asked the actors to show a neutral facial expression in order to achieve comparability between stimuli. As mentioned, given their communicative nature and familiarity, MF hand gestures usually occur with a facial expression (e.g., applauding or waving while smiling), which can provide a clue about the person’s intentions that may influence the person to engage in imitation or not (Vivanti et al., 2008). To control that the actors’ facial expressions did not differ between MF and ML hand gesture stimuli, we conducted an analysis of automated emotion recognition using the FER (Facial Emotion Recognition) Python package (https://ieeexplore.ieee.org/document/7553523). We extracted the individual frames of each video and identified the emotions represented in each frame. For each frame, the confidence for each of the seven emotions (anger, disgust, fear, happiness, neutral, sadness, and surprise) was predicted and used to identify the most dominant emotion (see Additional File 2). Using this frame-by-frame emotion distribution we found the mean emotion distribution for all frames in the video and identified the dominant emotion represented in each frame. Using this method, we found that for 7 of 8 videos, the neutral emotion had the highest mean confidence probability and was predominantly identified on average in all stimuli (see Additional file 2 for more details).

### Video Recordings

Testing took place at the Autism Brain and Behavior Lab in Geneva, Switzerland. Participants either sat on their own or on their caregivers’ laps approximately 60 cm from a 1920 × 1200 pixel screen. Gaze data were collected using Tobii Studio software 3.10.6 on a TX300 Tobii eye-tracker system with a 300 Hz sampling rate (Tobii Technology, Stockholm, Sweden). A 5-point calibration adapted for young children preceded the task. A static camera (Microsoft LifeCam Studio™) was attached at the top of the monitor to capture the child’s face and hands. Video recording was performed using The Observer XT™ software 14.2 for later behavioral coding (Noldus Information Technology. (2019). The Observer XT (Version 14.2). [Computer Software]. Retrieved 23 Apr 2022, from https://www.noldus.com/observer-xt). To synchronize the recording of gaze and imitation behavior, we launched the eye-tracking task from The Observer XT™ software.

### Analysis Strategy

As described in previous sections, to capture the processes behind imitation behavior, we focused on factors relating to visual attention (i.e., gaze data) and imitation performance (i.e., imitation data).

### Visual Attention

**Fig. 3:**
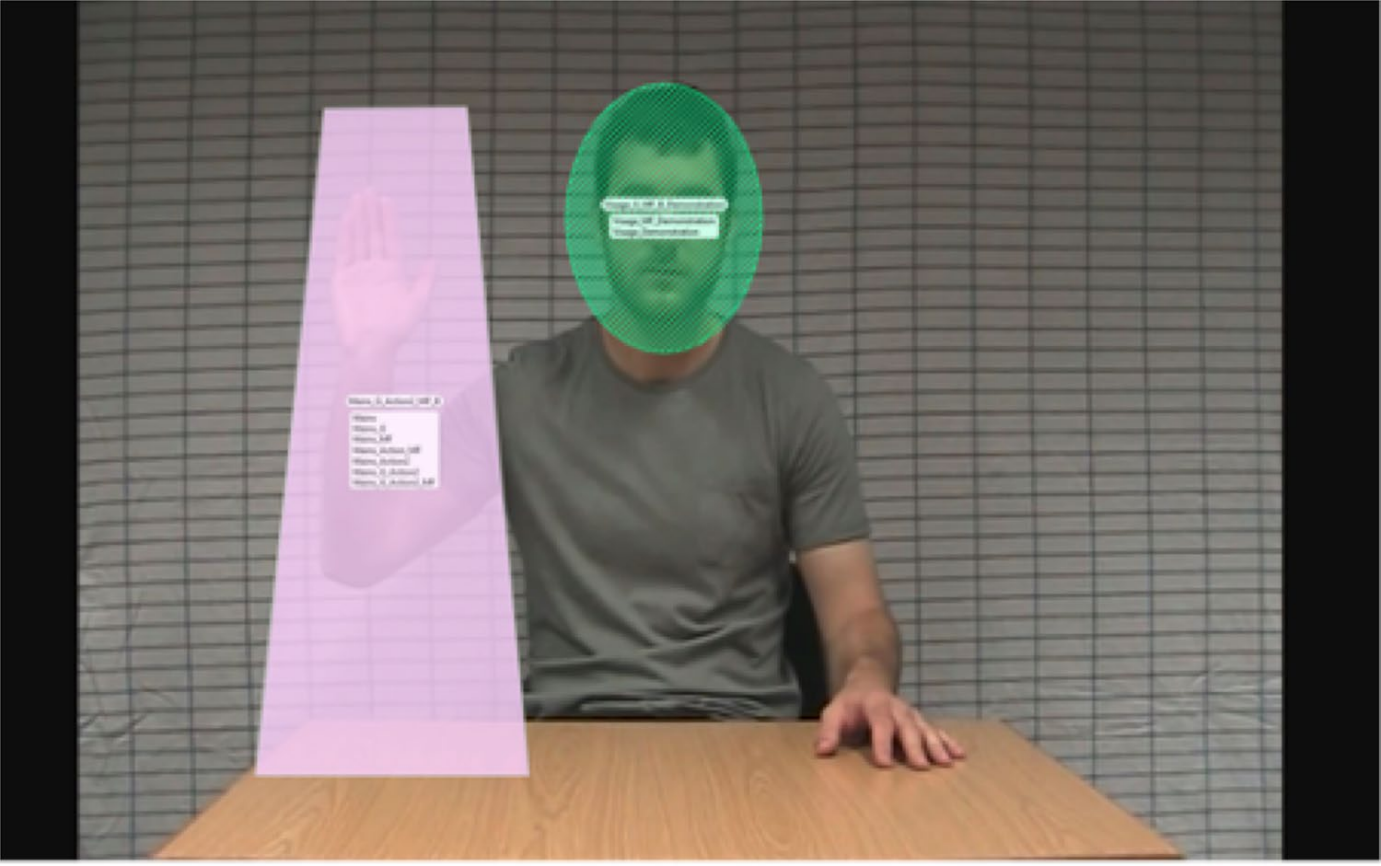
Eye-tracking stimuli and areas of interest around the face and the hand gesture

To investigate visual attention, we examined how children deployed their visual attention to the social cues, comprising both the actors’ directed speech (i.e., the green and gray dialog boxes in Fig. 2) and the demonstrations of hand and facial gestures (i.e., the red stars in Fig. 2). First, we examined the visual fixation time of children to the actors’ hands for MF and ML hand gestures and to the actors’ faces for FAC gestures during the three trials of each gesture (corresponding to the blue rectangles in Fig. 2), and also during the demonstrated actions specifically (corresponding to the orange rectangles in Fig. 2). To do this, we used Tobii Studio to draw areas of interest (AOIs). We drew dynamic AOIs around the actors’ hands (for MF and ML gestures) and faces for FAC gestures. The Face AOI was the same size across all stimuli, 376 pixels (9°28’) high and 270 pixels (6°48’) wide (Fig. 3). The measure of total fixation duration was defined as the sum of fixations within an AOI. Total fixation duration within an AOI was divided by the total time spent on the whole screen and multiplied by 100 to obtain the percentage of fixation duration for each AOI. We defined fixations with the IV-T Fixation filter on Tobii (Olsen, 2012) such that the minimum fixation duration was 60 ms. Secondly, we also examined whether visual attention toward gesture demonstrations was associated with imitation performance by conducting partial correlation analyses, controlling for age. Thirdly, we explored fixation duration to the actors’ faces during directed speech (i.e., when they asked the child to look at them and to imitate; see the dialog boxes in Fig. 2). We conducted t-tests to compare the visual exploration and imitation behavior of children in the ASD (*n*’s from 55 to 68) and TD (*n* = 15) groups. We used GraphPad PRISM (version 8.0.1 for Macintosh) to conduct these analyses and to plot graphs.

### Imitation Performance

To assess imitation behavior elicited by the actor’s invitations, all videos of children were scored using The Observer XT™ software. Imitation performance was analyzed both in terms of quantity and quality. To do so, we computed one *frequency* score and one *accuracy* score. First, a *frequency* score of 1 was assigned if the child partially or completely imitated the demonstrated gesture, whereas a *frequency* score of 0 was given if the child showed no imitation. Second, an *accuracy* score of 2 was assigned if the child’s imitation was precise and complete. A score of 1 was assigned if the child’s imitation was equivocal or incomplete (e.g., the child raised his hand but did not wave), and a score of 0 was given if the child showed no imitation. The maximum total frequency score was 9 (i.e., imitation in all trials) and the maximum accuracy score was 18 (i.e., accurate imitation in all trials). If a child imitated twice during the same trial, the best imitation attempt was coded. Imitation behaviors were coded by two independent raters (KL and PM). Interrater reliability was computed for both frequency and accuracy scores on 20% of the data set. Cohen’s kappas were 0.944 for the frequency score and 0.841 for the accuracy score.

To analyze imitation performance in the ASD and TD groups, we computed correlations between imitation scores and ages in each group. Then, we explored how the accuracy of imitation was related to the clinical profile of participants with ASD (i.e., autistic symptoms, developmental and adaptive skills) by conducting correlations controlling for age. To do so, we used MATLAB R2019b (MathWorks). *P*-values were corrected for multiple comparisons using the Benjamini–Hochberg false discovery rate (FDR) (Benjamini et al., 2001).

## Results

### Visual Attention

**Fig. 4:**
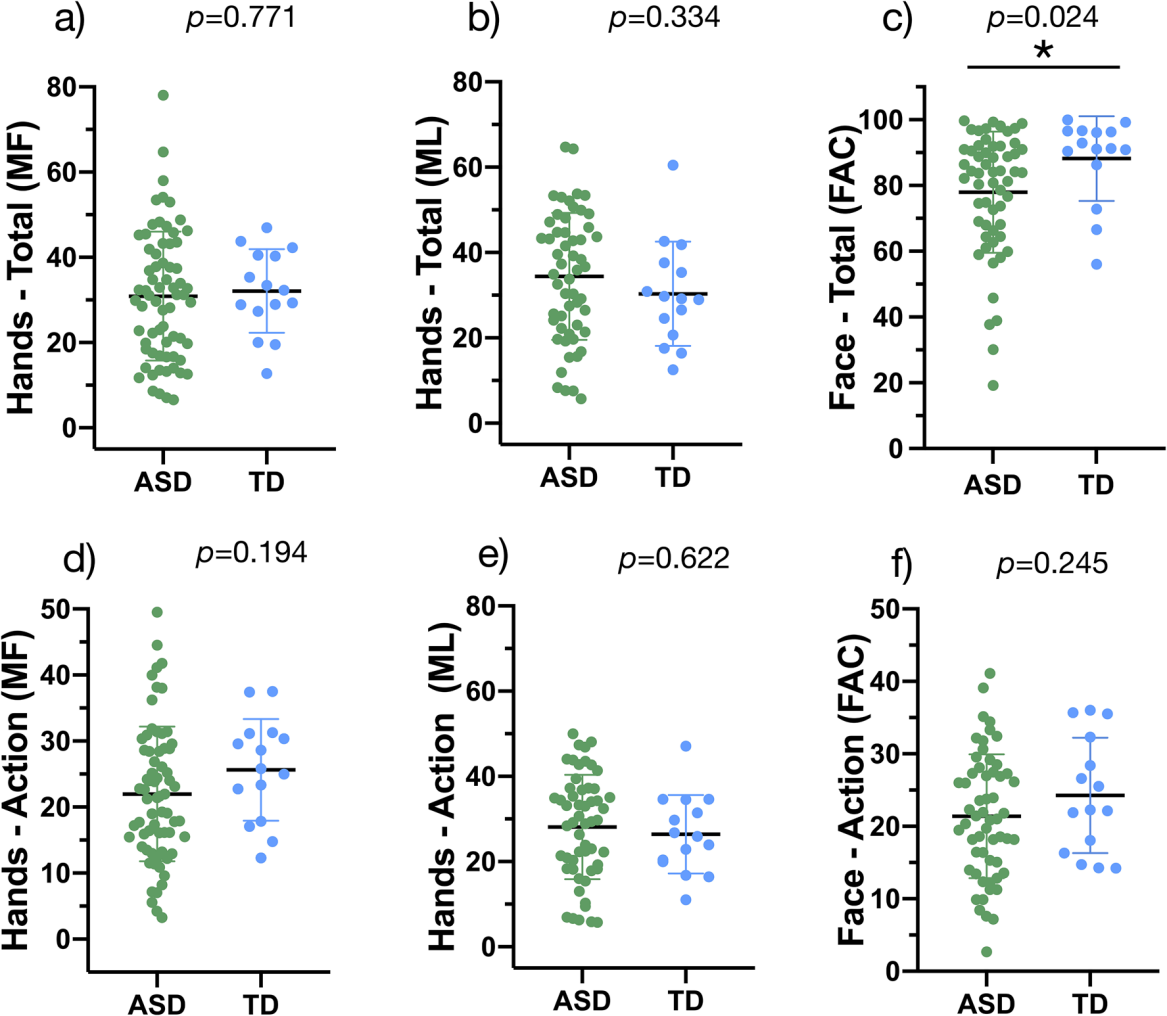
Percentage of fixation duration to the hands during MF (**a**) and ML (**b**) gestures, and to the face during FAC gestures (**c**). Percentage of fixation duration to the hands during demonstrations of MF (**d**) and ML (**e**) gestures, and to the face during demonstrations of FAC gestures (**f**)

When looking at the three trials for each gesture (i.e., the blue rectangles in Fig. 2) comparisons between the ASD and TD groups revealed no statistically significant difference in fixation duration to the hands during the MF (*M* = 30.9 s for the ASD group, and 32.1 s for the TD group, Fig. 4a) and ML hand gestures (*M* = 34.4 s for the ASD group, and 30.3 s for the TD group, Fig. 4b). However, our results showed that children with ASD spent less time than TD children looking at the actor’s face during FAC gestures (*M* = 78 s for the ASD group, and 88.2 s for the TD group, *p* = 0.024, Fig. 4c). Furthermore, when focusing only on the demonstrations of gestures (i.e., the red stars in Fig. 2), we found no significant difference between children with ASD and their TD peers in terms of fixation duration to the face during FAC gestures, and to the hands during MF and ML hand gestures (p > 0.05, Fig. 4d–f). Our results regarding FAC gestures suggest that while children with ASD looked less to the face overall, they still oriented to the face during crucial moments (i.e., demonstrations of FAC gestures).

**Fig. 5:**
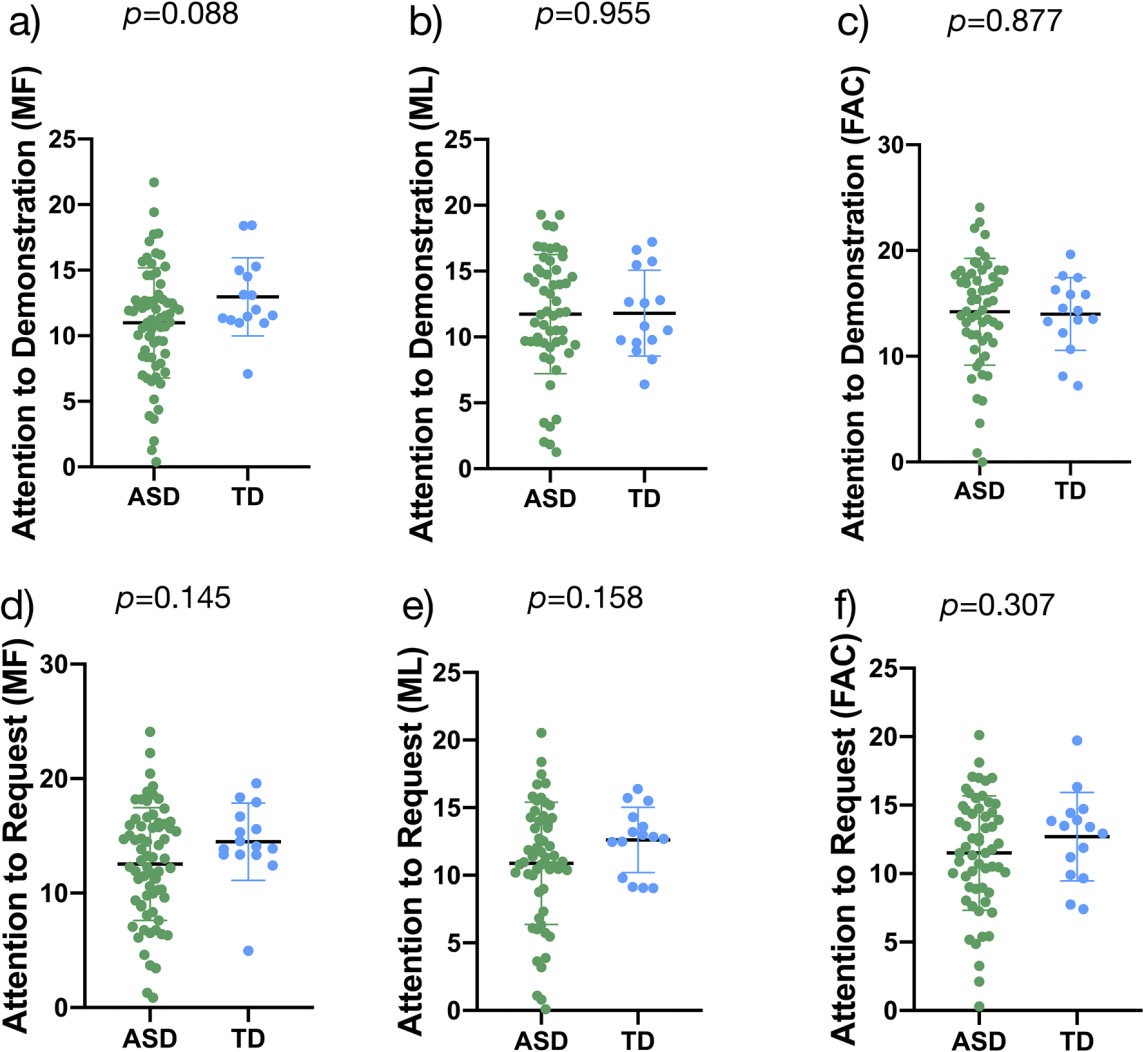
Percentage of fixation duration to the actors’ faces when asking the child to attend to the demonstration, for each type of gesture (**a, b, c**). Percentage of fixation duration to the actors’ faces when inviting the child to imitate for each type of gesture (**d, e, f**)

Concerning attention to the actors’ directed speech, we found no difference in fixation duration to the face during the demonstration and request between the ASD and TD groups (*p* > 0.05, Fig. 5). However, the distribution of fixation duration in the ASD group shows higher variability for all types of gestures.

### Imitation Performance

First, we compared the groups’ imitation frequency and accuracy scores for each type of gesture (MF, ML, FAC). Our results indicate no difference in any of the scores between the ASD and the TD groups (*p* > 0.05, Additional file 3). As a significant proportion of children in both groups never imitated (respectively, 29% in the ASD group and 46% in the TD group), we isolated those children (denoted “non-imitators”) to compare them with children with an imitation score of at least 1 (denoted “imitators”). In the ASD group, non-imitators and imitators had similar levels of autistic symptoms (*p* > 0.05) and developmental skills (measured by the four subscales DQ of the MSEL, *p* > 0.05, Additional file 4). However, imitators (*M* = 3.70) in the ASD group were older than non-imitators (*M* = 3.70, *p* = 0.024). As we hypothesized, we found significant Spearman correlations indicating that the total accuracy imitation score increases with chronological age in both groups (Additional file 5). Imitation scores were also significantly positively correlated with developmental age (Additional file 5).

Given our hypothesis that longer looking at the demonstrations of gestures would lead to better imitative behavior, we conducted partial correlations between these and controlled for age. In the ASD group, we found no significant correlations between frequency and accuracy scores of MF, ML, and FAC gestures and fixation duration to the demonstrations of MF, ML, and FAC gestures (Additional file 6). The absence of significant correlations suggests that fixation duration to gesture demonstrations was not associated with imitation behavior.

**Fig. 6:**
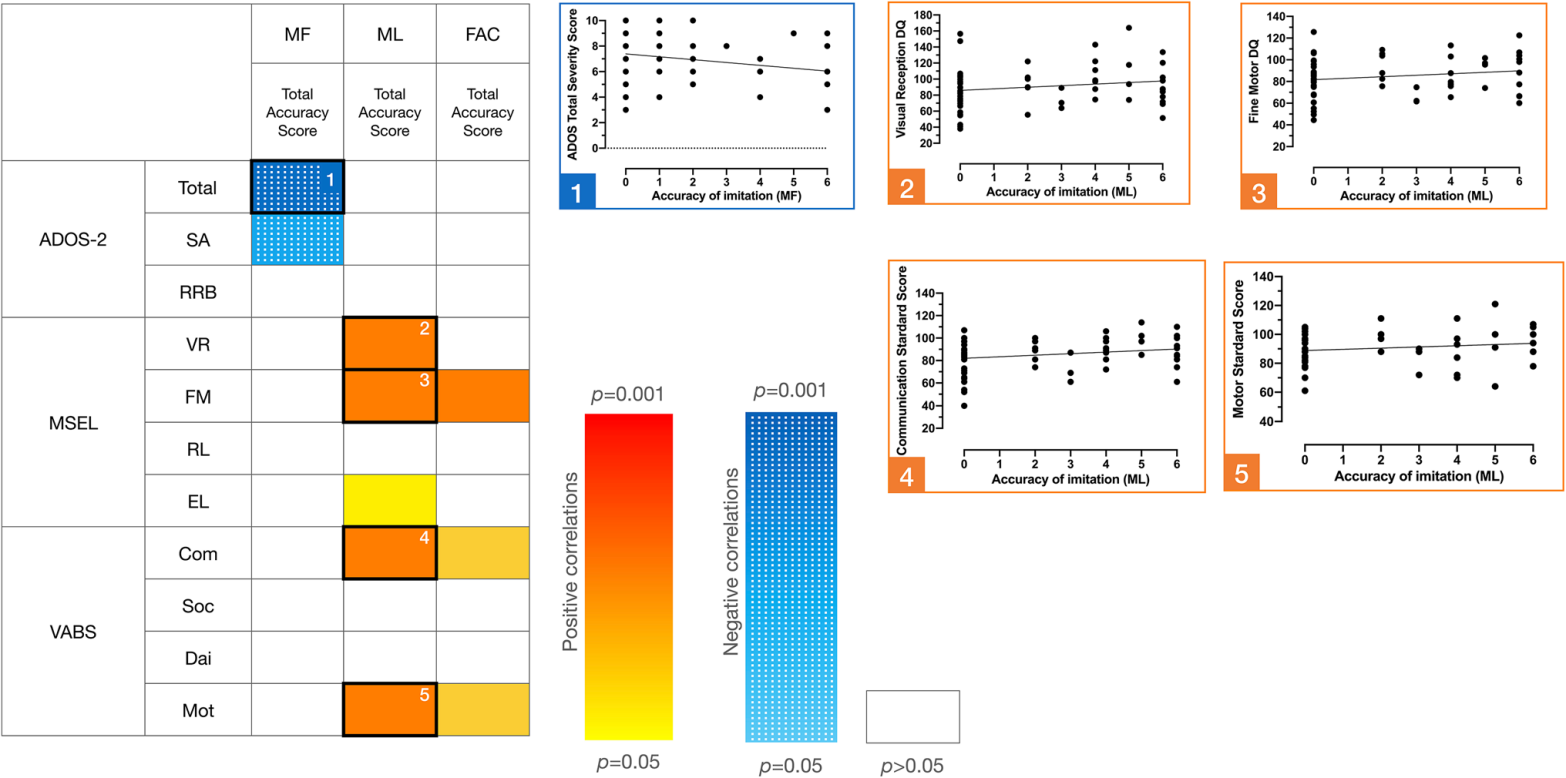
Correlation matrix between imitation accuracy scores for each type of gesture and clinical measures. ^1^ADOS-2, Autism Diagnostic Observation Schedule, 2nd edition; SA, Social Affect Severity Score; RRB, Restricted and Repetitive Behaviors; Total, Total Severity Score. ^2^MSEL, Mullen Scales of Early Learning; VR, Visual Reception; FM, Fine Motor; RL, Receptive Language; EL, Expressive Language. ^3^ VABS-II, Vineland Adaptive Behavior Scales, 2nd edition; Com, Communication standard score; Soc, Socialization standard score; Dai, Daily Living Skills standard score; Mot, Motor Skills standard score. The five correlations with black borders survive FDR correction and are plotted on the right

Moreover, we computed partial correlations between accuracy of imitation and ASD symptoms and developmental skills while controlling for age (Fig. 6 and Additional file 7). We applied an FDR correction for multiple comparisons. Our results show two patterns regarding hand gestures. First, imitation of MF gestures was negatively associated with autistic symptoms (*r* = -0.390, *p* = 0.001). In other words, children with lower levels of autistic symptoms demonstrated better imitation of MF gestures. We also found significant positive correlations between imitation of ML hand gestures and MSEL VR (*r* = 0.330, *p* = 0.009) and FM (*r* = 0.330, *p* = 0.008) domains, and VABS-II motor (*r* = 0.310, *p* = 0.014) and communication (*r* = 0.310, *p* = 0.015) skills. This result suggests that children with higher levels of visual and motor skills, and communication abilities imitate ML gestures better than those with lower skills. No significant correlations involving FAC gestures survived FDR correction (*p* > 0.05).

## Discussion

In this study, we investigated imitation behavior and related visual exploration in young children with ASD. To do this, we designed an imitation eye-tracking task during which we also acquired video recordings of the participants. We explored visual attention during demonstrations of meaningful and meaningless hand gestures, and facial gestures, during the child-directed speech of the actors demonstrating the gestures. In addition, we examined whether imitation performance of gestures was influenced by visual exploration and individual characteristics of the children (e.g., age, autistic symptoms, developmental skills). Regarding visual attention, we found that the ASD and TD groups displayed similar levels of visual attention toward gesture demonstrations and directed speech, although children with ASD spent less time fixating the face during facial (FAC) stimuli. Our results related to imitation behavior showed that (1) performance did not differ between the ASD and TD groups, but was correlated with chronological and developmental ages; (2) performance was not associated with attention to gesture demonstrations; and (3) imitation of meaningful (MF) hand gestures was associated with lower levels of autistic symptoms, whereas imitation of meaningless (ML) hand gestures was correlated with better-developed non-verbal cognitive skills and fine motor skills.

### Visual Attention

We investigated visual attention to a scene in which an actor demonstrated different types of gestures and then invited the participants to imitate. We aimed to explore how young children with ASD attended to demonstrations of gestures and to the actors’ child-directed speech, as well as the relation of these with imitation performance. First, we examined children’s visual exploration of the actors’ hands while demonstrating MF and ML gestures, and their faces during FAC gestures. We showed that children with ASD and their TD peers did not differ in looking at the hands during ML and MF gesture demonstrations. This result is in line with those of Vivanti and colleagues (Vivanti et al., 2008), who highlighted no eye-tracking differences in the action region for meaningless gestures and meaningful actions on objects between ASD and TD groups. Nevertheless, it should be noted that their ASD group was 8- to 15-year-olds without intellectual disability. Our result contrasts with those of Akin-Bulbul and Ozdemir (2022), who found diminished attention to the movement region of meaningful and meaningless actions on objects, gestures, and vocalizations in toddlers with ASD and those with TD. We argue that this discrepancy may be due to the types of imitation that were averaged by Akin-Bulbul and Ozdemir (2022). Although their ASD sample is similar to ours in terms of age and functioning level, comparison with their findings is compromised by their clustering of heterogeneous types of imitation. Indeed, heterogeneity and contradictory results across studies are frequent in research on imitation, illustrating the complexity of imitation (Rogers et al., 2006; Vivanti et al., 2008).

Furthermore, we found that young children with ASD displayed decreased visual attention to the actors’ face during FAC stimuli. This result is consistent with many previous studies of children with ASD (Akin-Bulbul & Ozdemir, 2022; Chawarska et al., 2013; Robain et al., 2021; Shic et al., 2014, 2020; Vivanti et al., 2008, 2014). In the context of an imitation task, looking at the actors’ faces is important as it provides crucial social information and intention about the performed gesture (Akin-Bulbul & Ozdemir, 2022; Vivanti et al., 2008). However, the ASD and TD groups showed similar looking time to the face during demonstrations of FAC gestures (Fig. 4f). This suggests that, although young children with ASD generally displayed less orientation to the face, they oriented to the salient parts of the task (i.e., facial movements). This finding echoes the results of Franchini et al. (2017), who demonstrated that salient facial expression (i.e., intense surprise) facilitated response to joint attention in preschoolers with ASD. Moreover, Vivanti et al. (2008) advanced three hypotheses as to why children with ASD spend less time looking at the face. Their first proposition is based on the social motivation theory (Chevallier et al., 2012; Dawson, 2008), and implies that young children with ASD orient less toward social stimuli, which reduces social learning experiences and negatively affects development of social skills. Their second proposition is that children with ASD assess social stimuli as threatening, and thus avoid them. Finally, their third proposition is related to difficulties in attention shifting between social and non-social stimuli that have been reported in ASD (Bryson et al., 2008; Landry & Bryson, 2004). According to this hypothesis, it is more difficult for individuals with ASD to disengage their attention from a stimulus and shift between two stimuli (Vivanti et al., 2008).

Our data do not support any of these hypotheses, as we only found a difference in orientation to face for FAC stimuli, and not MF and ML gestures (Fig. 4). Rather, we hypothesize that this difference may be due to the higher social demands during FAC stimuli. In other words, as the actors’ faces were central in FAC stimuli, and hands were not involved, focusing continuously on faces may have been difficult for children with ASD, particularly during child-directed speech when the actors’ facial expressions were neutral. In line with Franchini et al. (2017) we argue that rendering the face more salient with communicative cues (e.g., exaggerated facial expression or gestural pointing) helps young children with ASD with social engagement and attention-sharing behaviors. This finding is important as capturing the children’s attention with exaggerated cues is a core early intervention strategy for ASD (Franchini et al., 2017; Schreibman et al., 2015). Indeed, teaching such behaviors is critical as they support initiation and response to joint attention, and to a larger extent the development of social communication (Dawson et al., 2004; Franchini et al., 2017; Poon et al., 2012).

### Imitation Performance

Contrary to our hypothesis, our findings revealed that imitation scores did not differ between children with ASD and their TD peers on three types of gestures (MF, ML, and FAC). Although most studies (DeMyer et al., 1972; Rogers et al., 2003; Stone et al., 1990, 1997) have reported a specific imitation deficit in ASD, it is important to note that some studies (Charman & Baron-Cohen, 1994; Morgan et al., 1989) did not find impaired imitation skills in children with ASD. This inconsistent pattern may result from the different imitation tasks administered on samples that varied both in age and functioning level across studies. Nevertheless, it would be wrong to infer from our result that children with ASD do not have any difficulty in imitation. Indeed, the sample size of our TD group is too limited for generalization, and it is likely that additional factors affected the imitation scores of TD participants. For instance, it is likely that TD participants noticed the awkwardness of the situation (i.e., a video recording of a neutral stranger inviting them to copy gestures) which may also have reduced cooperation. Future studies need to assess these additional factors to quantify unwillingness versus incapacity to imitate in such experimental settings.

When examining individual factors related to imitation performance, we found that chronological and developmental age were positively associated with imitation scores. This association underlines that imitation is a developmental process (Nielsen et al., 2006; Vivanti & Hamilton, 2014) as children with more verbal and non-verbal skills performed better. One major finding of the present study is the distinct patterns observed between MF and ML hand gestures. From a conceptual standpoint, the difference between meaningful and meaningless actions relates to familiarity. MF gestures (e.g., waving, clapping, asking for silence, sending a kiss) are frequently performed in children’s everyday life while meaningless actions are novel (Vivanti & Hamilton, 2014). Moreover, MF gestures are symbolic and language-related actions while ML gestures have no semantic associations (Vanvuchelen et al., 2007). For this reason, Vivanti and Hamilton (2014) argue that copying of meaningless actions might reflect “true” imitation.

Our results support the position that children with ASD with better-developed nonverbal cognition and fine motor skills showed higher accuracy when imitating ML gestures. In other words, these skills are necessary to process visual information (i.e., the demonstrated actions) and execute the gesture. Further, we found that accuracy of imitating MF gestures was negatively correlated with autism symptom severity. This result ties well with the findings of Akin-Bulbul and Ozdemir (2022) who demonstrated that children with TD and DD imitated gestures better when they were meaningful but did not find the same facilitative effect of meaning for the ASD group. Therefore, it appears that imitating MF gestures accurately is more challenging for children with more severe autistic symptoms. In line with social motivation theory of imitation (Chevallier et al., 2012; Dawson, 2008), we infer that the more affected children may show less social interest in their environment and the people surrounding them. Consequently, by paying less attention to their environments, children with ASD benefit less from social learning and are less familiar with common gestures (Vivanti & Hamilton, 2014).

### Limitations and Perspectives

Overall, the present study contributes to a better understanding of the complexity of gesture imitation. By exploring the imitation of MF and ML gestures, we underlined their specific natures and the need to include both in imitation assessment and early intervention programs. The main limitation of the present study is the small TD group. Moreover, a significant proportion of TD participants never tried to imitate, possibly due to children’s shyness or task awkwardness as discussed. Future studies could employ naturalistic paradigms, such as contingency-based eye-tracking tasks, or using eye-tracking glasses in live-imitation tasks to enhance motivation in TD participants. In addition, future studies should continue to characterize children’s performance using imitation of different types of actions (e.g., gestures, actions on objects, meaningful, meaningless) with larger samples. Such investigations would allow to more clearly delineate imitation skills and deficits in young children with ASD. Given the heterogeneous ASD phenotype, it would be particularly valuable to distinguish profiles of children that are associated with specific patterns of imitation performance.

## Conclusion

In the present study, we showed that young children with ASD did not display atypical visual attention during an elicited imitation task, compared to TD peers. However, young children with ASD spent less time looking at the actor’s face when social demands were higher. Moreover, we found no correlation between visual exploration of the actor and imitation performance. Age and developmental skills were important influences on imitation performance in both ASD and TD groups. Finally, we highlighted the distinct nature of MF and ML gestures, revealing the complex nature of imitation.

Some clinical implications can be drawn from our findings. First, we argue that including gesture imitation in intervention programs is essential given the increased difficulty reported for children with autism. It should also be noted that imitation of actions with objects may also be a skill lacking for the more developmentally delayed children with autism. Second, we highlight that imitation of meaningful and meaningless gestures is a powerful tool for early intervention. Specifically, given that meaningful gestures have semantic content, they may support the development of language and vice versa. Teaching meaningful and meaningless gestures is essential to support both social and communication skills, and non-verbal cognitive skills.

## Supplementary Information

Below is the link to the electronic supplementary material.

Supplementary file1 (DOCX 499 KB)

## Data Availability

The datasets used in the current study are available from the corresponding author on reasonable request.
