# Supplementary material for: Gesture Imitation Performance and Visual Exploration in Young Children with Autism Spectrum Disorder
Source: J Autism Dev Disord. 2024 Oct 15;56(2):695–708. doi: 10.1007/s10803-024-06595-w (PMC12864208; doi:10.1007/s10803-024-06595-w)
Supplement: Supplementary file 1 — Supplementary file1 (DOCX 499 KB) [file 10803_2024_6595_MOESM1_ESM.docx]

# Supplementary Information

## Additional file 1

|  | **Initial sample (n=100)** | | |
| --- | --- | --- | --- |
|  | **ASD**  *n*=84 (14F) | **TD**  *n*=16 (7F) | ***p*** |
| **Age**  ***M(±SD)*** | 3.55 (±1.11) | 3.31 (±1.17) | 0.433 |
| **MSEL Total^1^** | 77.49 (±23.05) | 114.8 (±14.1) | <0.001 |
| **ADOS-2**  **Total^2^** | 6.92 (±1.74) | 1.27 (±0.59) | <0.001 |

Table A1. Cross-sectional sample demographics

##

## Additional file 2

### Analysis of automated emotion recognition

FER (Facial Emotion Recognition) is a python library based on face detection and alignment using multitask cascaded convolutional neural network (https://ieeexplore.ieee.org/document/7553523) and real-time convolutional neural network for emotion and gender classification (Arriaga et al., 2017). FER is an effective tool which carries out face detection alignment using multitask cascaded convolutional neural network and carries out a convolutional neural network-based emotion classification. For each of the video clips, we extract the individual frames in the form of PNG images and run the face detection and alignment convolutional neural network and emotion recognition convolutional neural network to detect the most dominant emotion among a variety of them (i.e., angriness, disgust, fear, happiness, sadness, surprise, and neutrality). The emotion detection convolutional neural network provides a confidence probability for the presence of each of the 7 emotions in cases where there is the presence of mixed emotions. We then use these emotion probabilities for individual frames and find the mean confidence probability across all the frames for a video clip to obtain the final mean confidence for each of the 7 emotions. Using this method, we estimate neutral emotions to have the highest mean confidence probability and were predominantly identified throughout all stimuli.

## Plots of analysis of automated emotion recognition

**
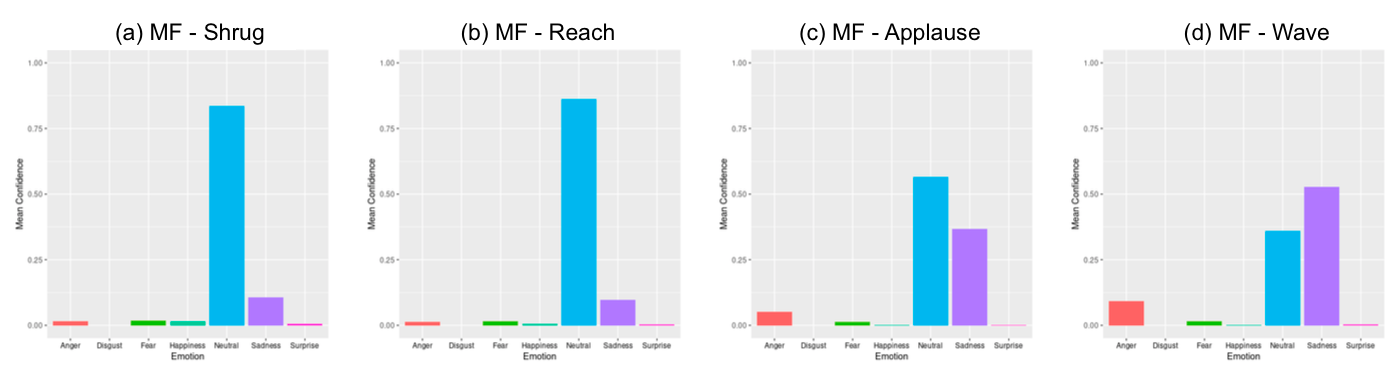
**

**
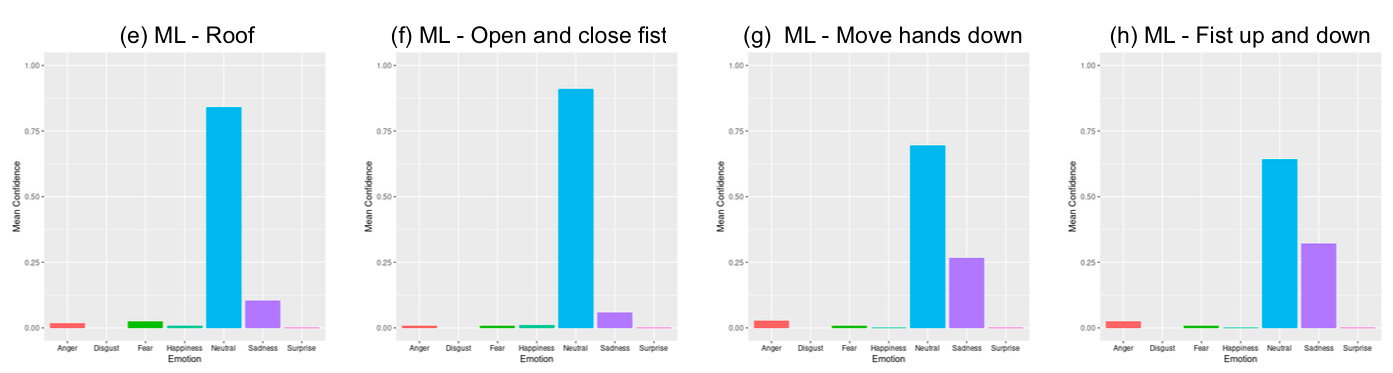
**

Figure A1. Histogram of automated emotion recognition for each gesture: (a) *Shrug* meaningful hand gesture; (b) *Reach* meaningful hand gesture; meaningful hand gesture; (c) *Applause* meaningful hand gesture; (d) *Wave* meaningful hand gesture; (e) *Roof* meaningless hand gesture; (f) *Open and close fist* meaningless hand gesture; (g) *Move hands down* meaningless hand gesture; (h) *Fist up and down* meaningless hand gesture

## Additional file 3

##
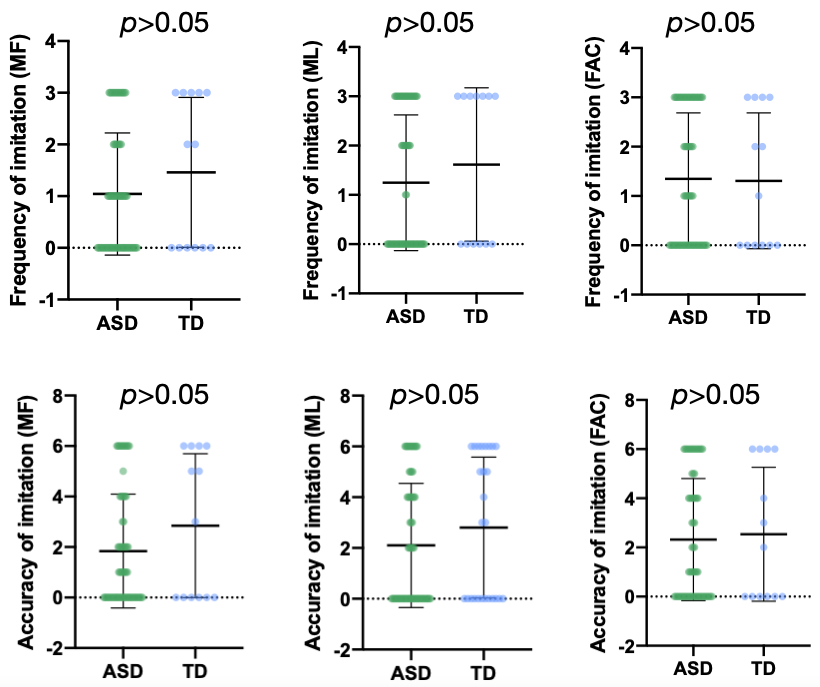


Figure A2. Comparison of imitation frequency and accuracy scores by type of gestures between the ASD and the TD groups.

| **Fixation duration [%] of…** | **ASD** (M ± SD) | **TD**  (M ± SD) | **t** | **df** | ***P*-value** |
| --- | --- | --- | --- | --- | --- |
| ***Hands during MF stimuli*** | 30.9, 15.2 | 32.1, 9.8 | 0.29 | 81 | 0.771 |
| ***Hands during ML stimuli*** | 34.4, 14.9 | 30.3, 12.2 | 0.97 | 68 | 0.334 |
| ***Hands during MF demonstrations*** | 22.0, 10.2 | 25.7, 7.7 | 1.31 | 81 | 0.194 |
| ***Hands during ML demonstrations*** | 28.1, 12.3 | 26.4, 9.2 | 0.50 | 68 | 0.622 |
| ***Face during FAC demonstrations*** | 21.4, 8.6 | 24.3, 8.0 | 1.17 | 70 | 0.245 |
| ***Face during Demonstration (MF)*** | 11.0, 4.2 | 13.0, 3.0 | 1.73 | 81 | 0.088 |
| ***Face during Demonstration (ML)*** | 11.7, 4.5 | 11.8, 3.3 | 0.06 | 68 | 0.955 |
| ***Face during Demonstration (FAC)*** | 14.2, 5.1 | 14.0, 3.4 | 0.15 | 70 | 0.877 |
| ***Face during Request (ML)*** | 10.9, 4.5 | 12.6, 2.4 | 1.43 | 68 | 0.158 |
| ***Face during Request (FAC)*** | 11.5, 4.2 | 12.7, 3.2 | 1.03 | 70 | 0.307 |

Table A2. Results of t-tests comparing the fixation duration of the ASD and TD groups on several Areas of Interest during the eye-tracking task. MF: meaningful hand gesture; ML: meaningless hand gesture; FAC: facial gesture.

| **Fixation duration [%] of…** | **ASD** (Median) | **TD**  (Median) | **U** | ***P*-value** |
| --- | --- | --- | --- | --- |
| ***Face during FAC stimuli*** | 83.88 | 91.29 | 265 | **0.024** |
| ***Face during Request (MF)*** | 12.94 | 14.09 | 386 | 0.145 |

Table A3. Results of Mann-Whitney U tests comparing the fixation duration of the ASD and TD groups on several Areas of Interest during the eye-tracking task. MF: meaningful hand gesture; ML: meaningless hand gesture; FAC: facial gesture.

## Additional file 4

|  | **ASD** | | |
| --- | --- | --- | --- |
|  | Imitators | Non-imitators | *p* |
| ***n*** | 45 | 19 |  |
| **Age** | 3.70 | 3.01 | 0.024 |
| **ADOS-2 Total Severity Score** | 6.91 | 6.84 | >0.05 |
| **ADOS-2 SA Severity Score** | 5.84 | 5,53 | >0.05 |
| **ADOS-2 RRB Severity Score** | 8.96 | 9.26 | >0.05 |
| **MSEL EL DQ** | 69.70 | 58.03 | >0.05 |
| **MSEL RL DQ** | 75.80 | 76.50 | >0.05 |
| **MSEL FM DQ** | 86.57 | 79.59 | >0.05 |
| **MSEL VR DQ** | 90.80 | 89.99 | >0.05 |

Table A4. Comparison of Imitators and Non-imitators in the ASD group.

|  | **TD** | | |
| --- | --- | --- | --- |
|  | Imitators | Non-imitators | *p* |
| ***n*** | 7 | 6 |  |
| **Age** | 3.83 ± 1.18 | 2.53 ± 0.82 | **0.039** |
| **MSEL EL DQ**  (M ± SD) | 99.14 ± 22.53 | 107.20 ± 19.87 | >0.05 |
| **MSEL RL DQ**  (M ± SD) | 123.40 ± 14.54 | 109.10 ± 18.67 | >0.05 |
| **MSEL FM DQ**  (M ± SD) | 114.30 ± 9.75 | 106.50 ± 12.12 | >0.05 |
| **MSEL VR DQ**  (M ± SD) | 130.80 ± 13.29 | 116.10 ± 20.79 | >0.05 |

Table A5. Comparison of Imitators and Non-imitators in the TD group.

|  | **ASD and TD** | | |
| --- | --- | --- | --- |
|  | Imitators | Non-imitators | *p* |
| ***n*** | 51 | 26 |  |
| **Age** | 3.72 ± 1.11 | 2.88 ± 1.00 | 0.002 |
| **MSEL EL DQ**  (M ± SD) | 75.95 ± 29.34 | 70.62 ± 30.50 | >0.05 |
| **MSEL RL DQ**  (M ± SD) | 82.56 ± 38.99 | 79.72 ± 30.20 | >0.05 |
| **MSEL FM DQ**  (M ± SD) | 88.94 ± 21.55 | 88.92 ± 18.64 | >0.05 |
| **MSEL VR DQ**  (M ± SD) | 101.0 ± 31.44 | 93.78 ± 25.89 | >0.05 |

Table A6. Comparison of Imitators and Non-imitators in ASD and TD groups.

## Additional file 5
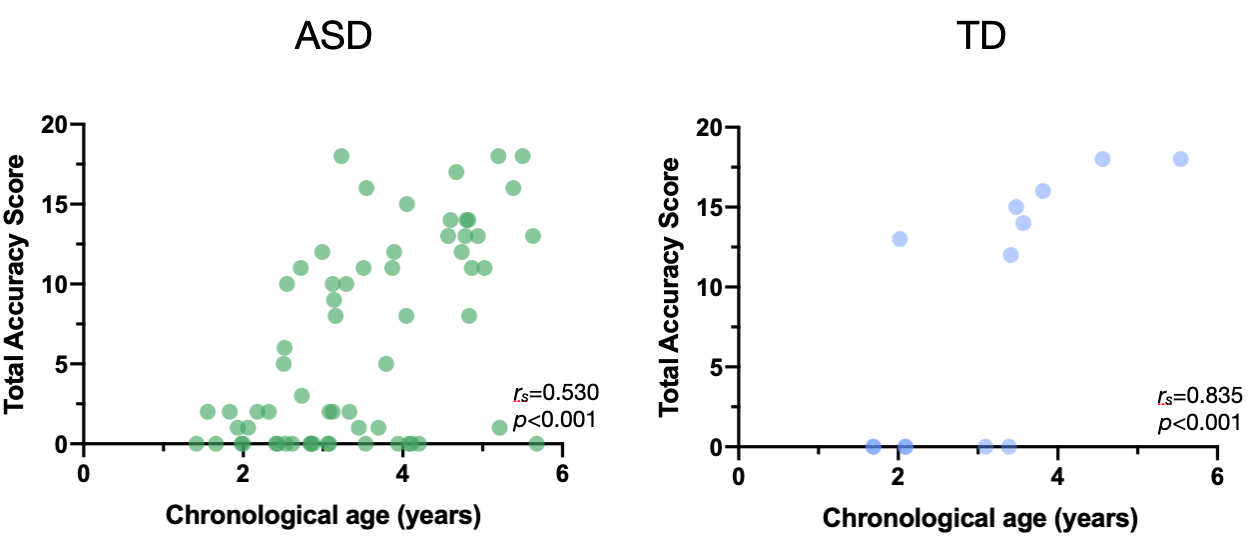


Figure A3. Correlation between chronological age and total accuracy imitation score.


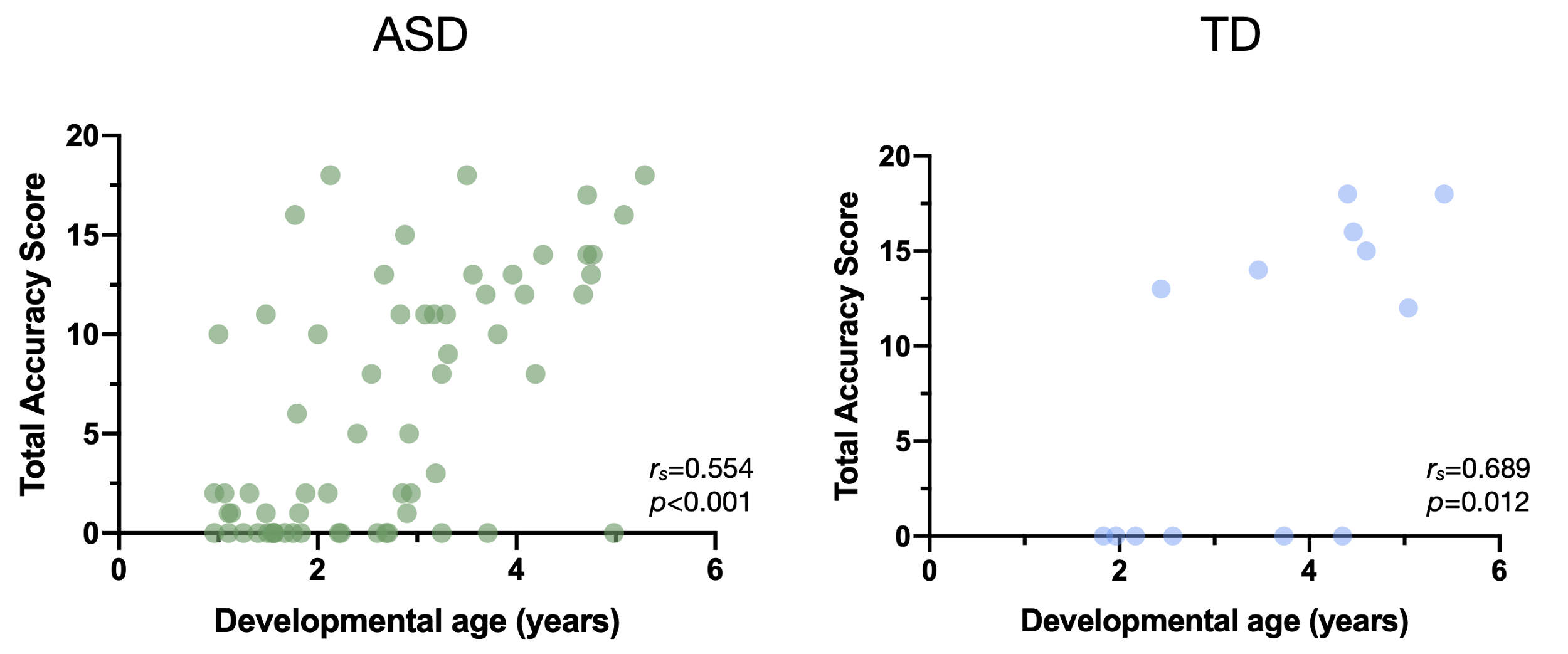


Figure A4. Correlation between developmental age and total accuracy imitation score.

## Additional file 6

|  |  | **Demonstrations of MF gestures** | **Demonstrations of ML gestures** | **Demonstrations of FAC gestures** |
| --- | --- | --- | --- | --- |
| **MF gestures** | *Frequency score* | *r_s_* = 0.125  *p* = 0.360 |  |  |
|  | *Accuracy score* | *r_s_* = 0.116  *p* = 0.393 |  |  |
| **ML gestures** | *Frequency score* |  | *r_s_* = 0.124  *p* = 0.416 |  |
|  | *Accuracy score* |  | *r_s_* = 0.04  *p* = 0.773 |  |
| **FAC gestures** | *Frequency score* |  |  | *r_s_* = 0.057  *p* = 0.705 |
|  | *Accuracy score* |  |  | *r_s_* = -0.043  *p* = 0.774 |

Table A7. Correlations between frequency and accuracy scores of MF, ML, and FAC gestures and fixation duration to the demonstrations of MF, ML, and FAC gestures.

## Additional file 7

|  | **Accuracy of imitation** | | |
| --- | --- | --- | --- |
|  | **MF hand gestures** | **ML hand gestures** | **FAC gestures** |
| **ADOS-2**  **SA**^1^ | *r* = -0.320  *p* = 0.011 | *r* = -0.110  *p* = 0.412 | *r* = 0.110  *p* = 0.391 |
| **ADOS-2**  **RRB**^1^ | *r* = 0.160  *p* = -0.213 | *r* = -0.110  *p* = 0.386 | *r* = -0.240  *p* = 0.056 |
| **ADOS-2**  **Total**^1^ | ***r* = -0.390**  ***p* = 0.001** | *r* = -0.100  *p* = 0.443 | *r* = 0.01  *p* = 0.937 |
| **MSEL**  **VR**^2^ | *r* = 0.140  *p* = 0.278 | ***r* = 0.330**  ***p* = 0.009** | *r* = 0.220  *p* = 0.089 |
| **MSEL**  **FM**^2^ | *r* = 0.210  *p* = 0.097 | ***r* = 0.330**  ***p* = 0.008** | *r* = 0.320  *p* = 0.010 |
| **MSEL**  **RL**^2^ | *r* = 0.160  *p* = 0.219 | *r* = 0.240  *p* = 0.059 | *r* = 0.210  *p* = 0.098 |
| **MSEL**  **EL**^2^ | *r* = 0.05  *p* = 0.697 | *r* = 0.250  *p* = 0.047 | *r* = 0.230  *p* = 0.075 |
| **VABS-II**  **Com**^3^ | *r* = 0.150  *p* = 0.232 | ***r* = 0.310**  ***p* = 0.014** | *r* = 0.300  *p* = 0.016 |
| **VABS-II**  **Soc**^3^ | *r* = 0.170  *p* = 0.176 | *r* = 0.210  *p* = 0.091 | *r* = 0.250  *p* = 0.046 |
| **VABS-II**  **Dai**^3^ | *r* = 0.230  *p* = 0.066 | *r* = 0.220  *p* = 0.087 | *r* = 0.290  *p* = 0.021 |
| **VABS-II**  **Mot**^3^ | *r* = 0.170  *p* = 0.195 | ***r* = 0.310**  ***p* = 0.015** | *r* = 0.270  *p* = 0.030 |

Table A8. Partial correlations between accuracy imitation scores for each type of gesture and clinical measures. ^1^ADOS-2, Autism Diagnostic Observation Schedule, 2^nd^ edition; SA, Social Affect Severity Score; RRB, Restricted and Repetitive Behaviors; Total, Total Severity Score. ^2^MSEL, Mullen Scales of Early Learning; VR, Visual Reception; FM, Fine Motor; RL, Receptive Language; EL, Expressive Language. ^3^ VABS-II, Vineland Adaptive Behavior Scales, 2^nd^ edition; Com, Communication Standard Score; Soc, Socialization Standard Score; Dai, Daily Living Skills Standard Score; Mot, Motor Skills Standard Score. Correlations in bold survive FDR correction.
